# Multichannel modulation of depolarizing and repolarizing ion currents increases the positive rate‐dependent action potential prolongation

**DOI:** 10.14814/phy2.15683

**Published:** 2023-05-05

**Authors:** Candido Cabo

**Affiliations:** ^1^ Department of Computer Systems, New York City College of Technology, Doctoral Program in Computer Science, Graduate Center City University of New York New York City New York USA

**Keywords:** computer models, ion channels, multichannel pharmacology, positive rate dependence

## Abstract

Prolongation of the action potential duration (APD) could prevent reentrant arrhythmias if prolongation occurs at the fast excitation rates of tachycardia with minimal prolongation at slow excitation rates (i.e., if prolongation is positive rate‐dependent). APD prolongation by current anti‐arrhythmic agents is either reverse (larger APD prolongation at slow rates than at fast rates) or neutral (similar APD prolongation at slow and fast rates), which may not result in an effective anti‐arrhythmic action. In this report we show that, in computer models of the human ventricular action potential, the combined modulation of both depolarizing and repolarizing ion currents results in a stronger positive rate‐dependent APD prolongation than modulation of repolarizing potassium currents. A robust positive rate‐dependent APD prolongation correlates with an acceleration of phase 2 repolarization and a deceleration of phase 3 repolarization, which leads to a triangulation of the action potential. A positive rate‐dependent APD prolongation decreases the repolarization reserve with respect to control, which can be managed by interventions that prolong APD at fast excitation rates and shorten APD at slow excitation rates. For both computer models of the action potential, I_CaL_ and I_K1_ are the most important ion currents to achieve a positive rate‐dependent APD prolongation. In conclusion, multichannel modulation of depolarizing and repolarizing ion currents, with ion channel activators and blockers, results in a robust APD prolongation at fast excitation rates, which should be anti‐arrhythmic, while minimizing APD prolongation at slow heart rates, which should reduce pro‐arrhythmic risks.

## Graphical abstract

The main conclusion of this computational study is that multichannel modulation of depolarizing and repolarizing ion currents, with ion channel activators and blockers, results in a robust action potential prolongation at fast excitation rates, which should be anti‐arrhythmic, while minimizing action potential prolongation at slow heart rates, which should reduce potential pro‐arrhythmic risks. These findings suggest the potential of multichannel pharmacology, with ion channel activators and blockers, as an antiarrhythmic strategy.

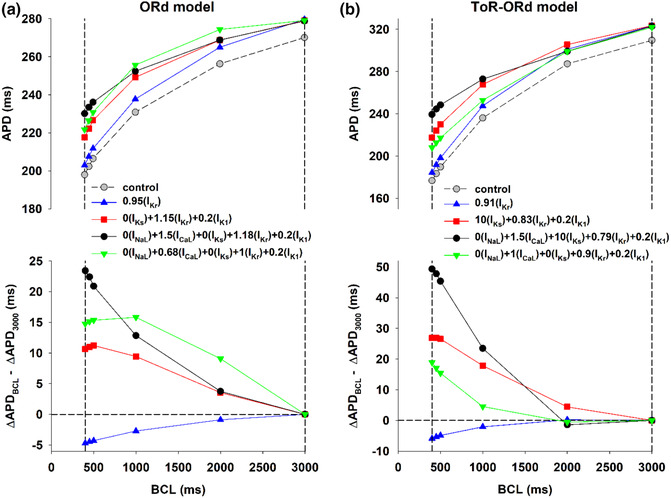

## 1 INTRODUCTION

A strategy for the treatment of reentrant tachyarrhythmias is the prolongation of the action potential duration (APD) using potassium channel blockers (Peters et al., 2000; Tamargo et al., 2004). However, clinical trials have shown that agents that selectively block potassium ion channels (like I_Kr_, the rapid delayed rectifier potassium current) are not effective at preventing initiation and maintenance of reentrant arrhythmias in post‐myocardial infarction patients (Bloch‐Thomsen, 1998; Køber et al., 2000; Waldo et al., 1996). APD prolongation with specific I_Kr_ blockers is reverse rate‐dependent, that is, APD prolongation is greater at slow excitation rates than at the fast excitation rates of ventricular tachycardia. That is far from optimal, not only because APD may not be sufficiently prolonged at the fast rates typical of ventricular tachycardia (Hondeghem & Snyders, 1990), but also because APD prolongation at slow rates may result in drug‐induced LQT syndrome, trigger early afterdepolarizations and have a pro‐arrhythmic effect (Kannankeril et al., 2010). For APD prolongation to be anti‐arrhythmic, APD prolongation should exhibit a positive rate‐dependent response, that is, it should maximize APD prolongation at fast excitation rates and minimize APD prolongation at slow excitation rates.

There is experimental and clinical evidence that multichannel pharmacology results in more favorable rate‐dependent APD prolongation and anti‐arrhythmic effects than pharmacology that selectively modulates a specific ion current. For example, verapamil derivatives like BRL‐32872, which block I_CaL_ (L‐type calcium current) and I_Kr_, show a neutral (i.e., neither positive nor reverse) rate‐dependent APD prolongation (Bril et al., 1995, 1998; Faivre et al., 1999; Nadler et al., 1998). The combination of class I/B (lidocaine, mexiletine) and class III (d‐sotalol) agents also has a favorable rate‐dependent response with antiarrhythmic effects (Lathrop & Varró, 1989, 1990; Mátyus et al., 2004; Varga et al., 2021). Inhibition of I_Ks_ and I_Kr_, both class III agents, also shows a neutral rate‐dependent APD prolongation (Qi et al., 1999; VerNooy & Mangrum, 2005). Amiodarone, an agent that is effective against a variety of clinical arrhythmias, prolongs APD by blocking sodium and potassium currents and also has an attenuated reverse rate dependence response when compared to agents that block selectively a specific ion current (Dorian & Newman, 2000; Hondeghem & Snyders, 1990). Consistent with that experimental and clinical evidence, we showed in an earlier report (Cabo, 2022) that, in computer models of the human ventricular action potential, APD prolongation by selectively blocking a specific potassium ion channel type results in a reverse or marginal positive rate dependence (Cabo, 2022). In contrast, APD prolongation by the combined modulation of several potassium ion currents (I_Ks_, I_Kr_ and I_K1_) can result in a robust positive rate dependence, suggesting the potential of multichannel pharmacology as an antiarrhythmic strategy (Cabo, 2022). In this report we further investigate how multichannel pharmacology can result in a robust positive rate‐dependent APD prolongation. We hypothesized that the combined modulation of depolarizing (I_NaL_ and I_CaL_) and repolarizing currents (I_Ks_, I_Kr_ and I_K1_) can further increase positive rate‐dependent APD prolongation. As before, we used computational models of the human ventricular action potential in combination with optimization algorithms to investigate which interventions may result in prolongation of APD with a positive rate dependence, and to further understand how the shape of the action potential relates to its rate dependence. We also investigated the relative importance of the different ion currents to achieve a positive rate‐dependent APD prolongation.

## 2 METHODS

### 2.1 Computer models of the action potential

We simulated the cardiac action potential using the ORd (O'Hara et al., 2011) and the ToR‐ORd (Tomek et al., 2019) models of a human ventricular epicardial cell. Both models are publicly available: the ORd model was downloaded from the Rudy Lab web site (https://rudylab.wustl.edu/code‐downloads/) and the ToR‐ORd model was downloaded from the CellML repository (www.cellml.org). The ToR‐ORd model builds on the structure of the ORd model, but the formulation of several currents that determine the rate dependence of the action potential, like I_CaL_, I_Kr_ and I_K1_, is different (Tomek et al., 2019).

We investigated the rate dependence of the action potential models by modulating the maximum conductance of two depolarizing currents: the late sodium current (I_NaL_; range: 0–2× control) and the L‐type calcium current (I_CaL_; range: 0.5–1.5× control); and three repolarizing currents: the slow delayed rectifier potassium current (I_Ks_; range: 0–20× control), the rapid delayed rectifier potassium current (I_Kr_; range: 0.5–2× control) and the inward rectifier potassium current (I_K1_; range: 0.2–2× control). Action potentials were initiated with a depolarizing current with a strength 1.5× the stimulation threshold. We report measurements on action potentials that were calculated after 30 min of stimulation to achieve steady‐state (Cabo, 2022).

### 2.2 Action potential features

To quantify and compare different action potential shapes we calculated the average ion current during phase 2 (I_ion,phase2_) and during phase 3 (I_ion,phase3_) as well as their ratio (I_ion,phase3_/I_ion,phase2_). The phases of the action potential were quantified as described in an earlier report (Cabo, 2022). In short, phase 1 begins at the time of action potential depolarization and it ends at the time repolarization starts, which is when the total ion current becomes positive (Figure 1 in Cabo, 2022). Phase 2 starts when phase 1 ends, and it ends when I_K1_ raises to 10% of its peak (Cabo, 2022). In the ORd model the end of phase 2 occurs when the membrane repolarizes to −39 mV. In the ToR‐ORd model the end of phase 2 occurs when the membrane repolarizes to −34 mV. Phase 3 starts at the end of phase 2, and it ends when the action potential repolarizes by 90% of the action potential amplitude from its maximum depolarization potential.

### 2.3 Estimation of the repolarization reserve

We estimated the repolarization reserve of a baseline action potential by quantifying the prolongation of the APD upon application of a constant depolarizing current of −0.1pA/pF during the action potential (Varro & Baczko, 2011). This can be done experimentally for example by increasing the late sodium current (I_NaL_) with veratrine and anemonia sulcata toxin (ATX II; Varro & Baczko, 2011). With that protocol, a larger prolongation of APD with respect to the baseline APD implies a smaller repolarization reserve and a higher risk of triggered arrhythmias.

### 2.4 Particle swarm optimization algorithm

When using selective potassium channel blockers to prolong the action potential, the prolongation of the action potential (with respect to control) at long cycle lengths (ΔAPD_long_) is generally larger than the prolongation of the action potential at short cycle lengths (ΔAPD_short_), resulting in reverse rate dependence (ΔAPD_long_ > ΔAPD_short_). As before (Cabo, 2022), we used the Particle Swarm Optimization (PSO) algorithm (Kennedy & Eberhart, 1995) to find the optimal combination of maximum conductance of I_NaL_, I_CaL_, I_Ks_, I_Kr_ and I_K1_ to minimize the difference between ΔAPD_long_ and ΔAPD_short_ while achieving a given APD prolongation. We used an implementation of the PSO algorithm publicly available in the Github repository (https://github.com/kkentzo/pso). Minimizing ΔAPD_long_‐ΔAPD_short_ should result in an attenuation of reverse rate dependence or achieving positive rate dependence if ΔAPD_long_ < ΔAPD_short_. In the simulations presented here, for both the ORd and ToR‐ORd model, the long cycle length was BCL = 3000 ms, and the short cycle length was BCL = 400 ms. The input of the PSO optimization algorithm was the APD goal and the allowed range of variation (minimum and maximum values) of ion channel maximum conductances.

### 2.5 Backward feature elimination

We used a backward feature elimination procedure to investigate the relative contribution of each ion current to the action potential positive rate dependence response. After applying the PSO optimization to find the combination of maximum conductances of I_NaL_, I_CaL_, I_Ks_, I_Kr_ and I_K1_ that maximize a positive rate dependence response, optimization was applied to the five possible subsets of four currents (i.e., [I_CaL_, I_Ks_, I_Kr_ and I_K1_], [I_NaL_, I_Ks_, I_Kr_ and I_K1_], [I_NaL_, I_CaL_, I_Kr_ and I_K1_], [I_NaL_, I_CaL_, I_Ks_ and I_K1_], [I_NaL_, I_CaL_, I_Ks_ and I_Kr_]). The subset resulting in the larger positive rate dependence response after PSO optimization was selected for the next step in the elimination procedure. The ion current not present in the selected subset was the current that contributed less to the positive rate dependence response and it was consequently eliminated (i.e., its maximum conductance was set to the control value). This process of elimination was repeated until only one ion current was left.

## 3 RESULTS

### 3.1 Positive rate dependence with multi‐channel pharmacology

**FIGURE 1:**
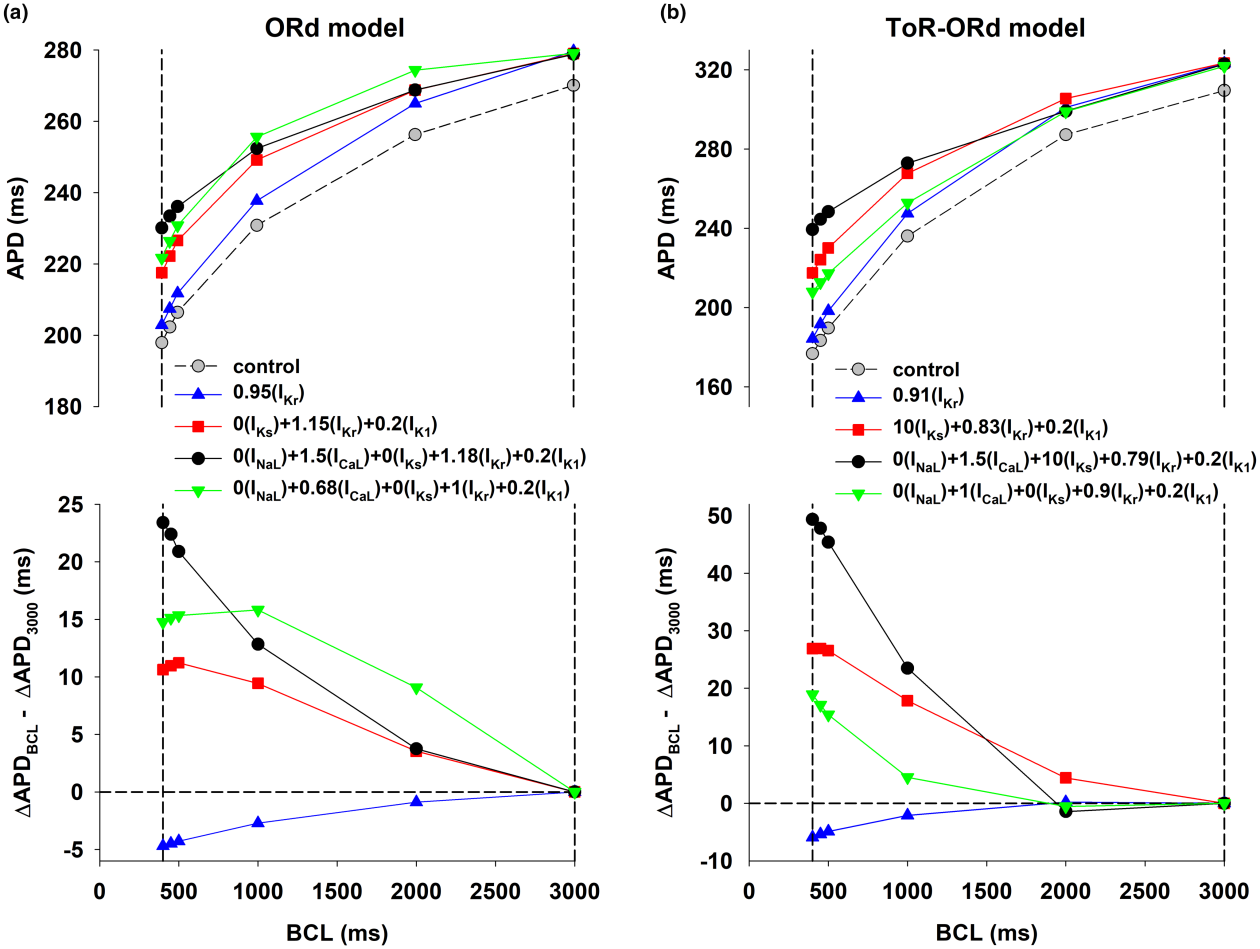
Rate dependence of APD prolongation with the ORd (a) and ToR‐ORd (b) models. Panel a (top): APD rate dependence during control (gray circles), and for four interventions that prolonged the control APD to 280 ms when BCL = 3000 ms, using the ORd model. The vertical dashed lines indicate BCL = 400 and 3000 ms respectively. Panel a (bottom): Difference between APD prolongation (with respect to control) for each BCL and APD prolongation when BCL = 3000 ms for the four interventions in Panel a (top). Positive values indicate positive rate dependence and negative values indicate reverse rate dependence. Panel b shows similar results for four interventions that prolong the control APD to 320 ms, when BCL = 3000 ms, using the ToR‐ORd model. The format is the same as in panel a. See text for detailed description.

Figure 1a (top) shows the APD rate dependence during control (gray circles, dashed line), and for four interventions that prolonged the control APD to 280 ms when BCL = 3000 ms, using the ORd model. While APD prolongation at BCL = 3000 ms (right vertical dashed line) is the same for all interventions (a prolongation of ~10 ms with respect to control), APD prolongation for shorter BCLs was markedly different (left vertical dashed line), indicating a different APD rate dependence. Figure 1a (bottom) shows how APD prolongation with respect to control at different BCLs compares to APD prolongation at BCL = 3000 ms for the four interventions. A positive value indicates that APD prolongation at a given BCL is larger that APD prolongation at 3000 ms, and shows positive rate dependence; a negative value indicates reverse rate dependence. The optimal combinations of ion channel activators and/or blockers were obtained using the PSO optimization algorithm described in Section 2.4.

It is well established experimentally and computationally that prolonging APD by blocking I_Kr_ (Figure 1a, blue triangles up) results in a reverse rate dependent response (Banyasz et al., 2009; Barandi et al., 2010; Cabo, 2022; Dorian & Newman, 2000; Virag et al., 2009). However, by modulating repolarizing potassium currents (I_Ks_, I_Kr_, and I_K1_), it is possible to achieve a positive rate dependent response (Figure 1, red squares; Cabo, 2022). Moreover, Figure 1a shows that modulation of both repolarizing currents (I_Ks_, I_Kr_, and I_K1_) and depolarizing currents (I_NaL_ and I_CaL_; Figure 1, black circles) drastically enhances the positive rate‐dependent APD prolongation at faster rates (i.e., BCLs <500 ms). Figure 1a also shows that modulation of depolarizing and repolarizing currents with ion channel activators and blockers (Figure 1, black circles) results in a more robust positive rate‐dependent APD prolongation than modulation of the same currents with only ion channel blockers at BCLs <500 ms (Figure 1, green triangles down).

Figure 1b shows similar results for interventions that prolong APD to 320 ms using the ToR‐ORd model (a prolongation of ~10 ms with respect to control). Note that there are quantitative differences between both models, a consequence of the different formulation of I_CaL_, I_Kr_ and I_K1_. For example, APD during control when BCL = 3000 ms is larger for the ToR‐ORd model (310 ms) than for the ORd model (270 ms). In contrast, the control APD of the ToR‐ORd model when BCL = 400 ms is shorter than that of the ORd model, which indicates a larger APD rate adaptation of the ToR‐ORd model. The larger *y*‐axis values in Figure 1a,b (bottom) also show that the positive rate dependence response is more robust for the ToR‐ORd model than for the ORd model.

Despite quantitative differences between the models, the results in Figure 1 show that modulating both depolarizing and repolarizing ion currents (Figure 1, black circles) results in a stronger positive rate dependence than modulation of just repolarizing potassium currents (Figure 1, red squares). Moreover, modulation of ion currents with ion channel activators and blockers (Figure 1, black circles) produces a more robust positive rate dependent APD prolongation than modulation of the same ion currents with only ion channel blockers (Figure 1, green triangles down). Modulation of a single repolarizing current (I_Kr_) results in reverse rate dependence.

### 3.2 Action potential shape and its effect on a positive rate dependence

**FIGURE 2:**
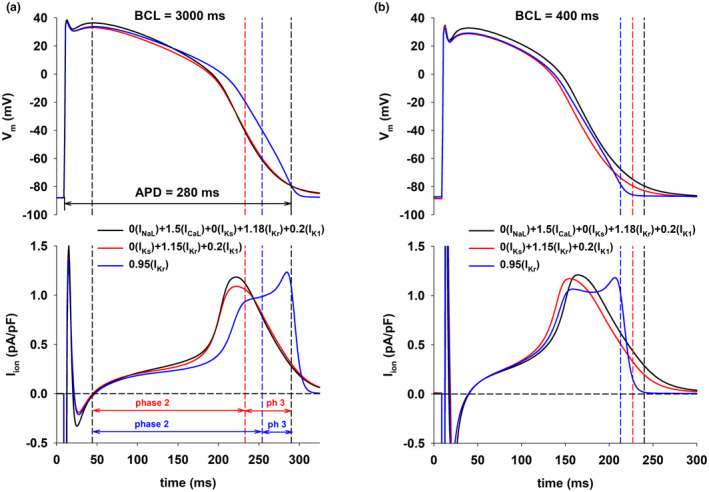
Panel (a) Action potentials for different interventions that prolong the action potential to 280 ms when BCL = 3000 ms (top) with the corresponding total ion currents (bottom), using the ORd model. The leftmost vertical black dashed line indicates the beginning of phase 2 of the action potential. The vertical red dashed line indicates the end of phase 2 for the (red and black) action potentials with a positive rate dependence. The vertical blue dashed line indicates the end of phase 2 for the (blue) action potential with a reverse rate dependence. The rightmost vertical black dashed line indicates the time of 90% repolarization, which is the same for the three action potentials. Phase 3 (ph 3) is the interval between the end of phase 2 and the time of 90% repolarization. Panel b: Action potentials when BCL = 400 ms for the interventions in panel a (top) with the corresponding total ion currents (bottom). The dashed (color coded) vertical lines indicate the time of 90% repolarization for the three interventions. See text for detailed description.

Figure 2a (top) shows action potentials for three of the interventions in Figure 1a that resulted in APD = 280 ms with BCL = 3000 ms, a prolongation of about 10 ms the control APD, using the ORd model. The action potentials are superimposed to make it easier to compare their shapes. Figure 2a (bottom) shows the corresponding total ion currents (I_ion_) during the action potentials. Figure 2a also shows the duration of phase 2 and phase 3 for the different action potentials (red and blue vertical dashed lines). Phase 2 starts at the leftmost vertical dashed line (Figure 2a) and ends when action potentials repolarize to −39 mV (red and blue vertical dashed lines). The end of phase 2 for the action potentials with multichannel modulation (black and red action potentials in Figure 2a) is the same. Phase 3 begins at the end of phase 2 and ends at the time of 90% repolarization (rightmost vertical dashed line in Figure 2a); the end of phase 3 is the same for all action potentials.

Interventions that result in a positive rate dependence (red and black lines in Figure 2a, bottom) have a larger I_ion_ during early action potential repolarization than the intervention that results in reverse rate dependence (blue line in Figure 2a, bottom). In contrast, during late repolarization of the action potential, I_ion_ is larger for the intervention that results in reverse rate dependence than for interventions that result in a positive rate dependence. This is reflected in a shorter phase 2 (and a longer phase 3) for interventions that result in positive rate dependence (Figure 2a, bottom).

The duration of phase 2 and phase 3 for the two interventions that result in a positive rate dependence is about the same (red and black action potentials in Figure 2a). But note that I_ion_ during phase 2 is larger for the intervention that modulated *both* depolarizing and repolarizing currents and that resulted in a larger positive rate dependent response (compare black and red lines in Figure 2a, bottom, during phase 2); this is a consequence of the larger depolarization produced by the 50% increase in I_CaL_ at the beginning of phase 2. The total I_ion_ for the interventions that result in a positive rate dependence (black and red lines in Figure 2a, bottom) during phase 3 is about the same, which is consistent with the fact that I_K1_, the largest current during late repolarization, is blocked by the same amount (80%) for both interventions.

Figure 2b (top) shows the corresponding action potentials for the three interventions when BCL = 400 ms. The vertical dashed lines indicate the time of 90% repolarization for the different action potentials to illustrate the differences in APD for the three interventions at the faster stimulation rate. Figure 2b (bottom) shows the corresponding total ion currents. The results are qualitatively similar to those in Figure 2a, that is, positive rate dependence is associated with faster repolarization during phase 2 and slower repolarization during phase 3 of the action potential. Overall, the results in Figure 2 show that, in the ORd model, a positive rate‐dependent response is associated with a larger I_ion_ (i.e., a faster repolarization) during phase 2 and a smaller I_ion_ (i.e., a slower repolarization) during phase 3 of the action potential.

**FIGURE 3:**
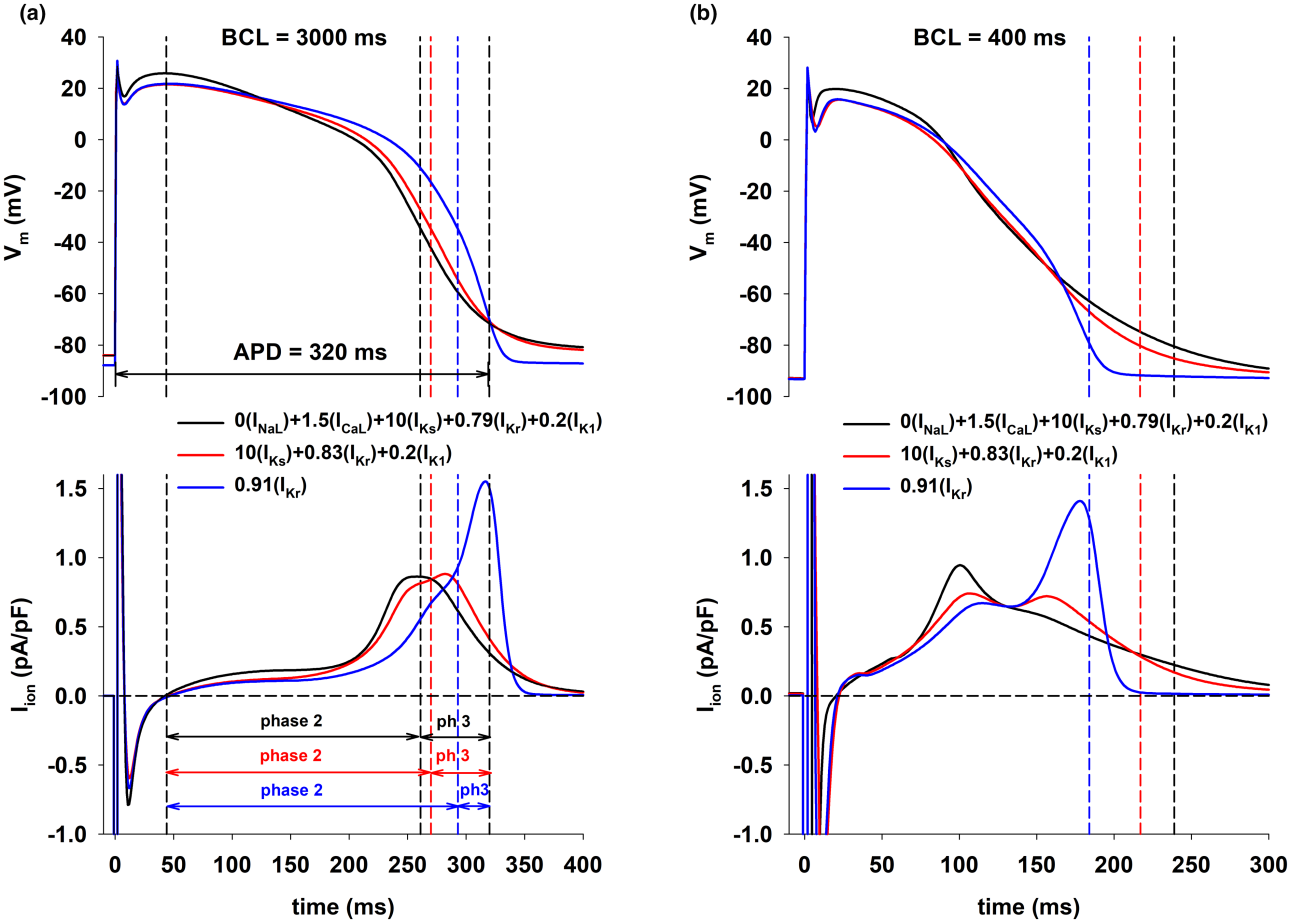
Panel a: Action potentials for different interventions that prolong the action potential to 320 ms when BCL = 3000 ms (top) with the corresponding total ion currents (bottom), using the ToR‐ORd model. The leftmost vertical black dashed line indicates the beginning of phase 2 of the action potential. The vertical red/black dashed line indicate the end of phase 2 for the red/black action potential with a positive rate dependence. The vertical blue dashed line indicates the end of phase 2 for the blue action potential with a reverse rate dependence. The rightmost vertical black dashed line indicates the time of 90% repolarization, which is the same for the three action potentials. Phase 3 (ph 3) is the interval between the end of phase 2 and the time of 90% repolarization. Panel b: Action potentials when BCL = 400 ms for the interventions in panel a (top) with the corresponding total ion currents (bottom). The dashed (color coded) vertical lines indicate the time of 90% repolarization for the three interventions. See text for detailed description.

Figure 3 (top) shows action potentials for three of the interventions in Figure 1b with BCL = 3000 ms (Figure 3a) and BCL = 400 ms (Figure 3b), using the ToR‐ORd model. Figure 3 (bottom) shows the corresponding total ion currents (I_ion_) during the action potentials. The figure format is the same as in Figure 2. Note that in spite of the quantitative differences in the formulation of the models, the results obtained with the ORd model (Figure 2) and the ToR‐ORd model (Figure 3) are similar: a positive rate‐dependent response is associated with a larger I_ion_ (i.e., a faster repolarization) during phase 2 and a smaller I_ion_ (i.e., a slower repolarization) during phase 3 of the action potential.

**FIGURE 4:**
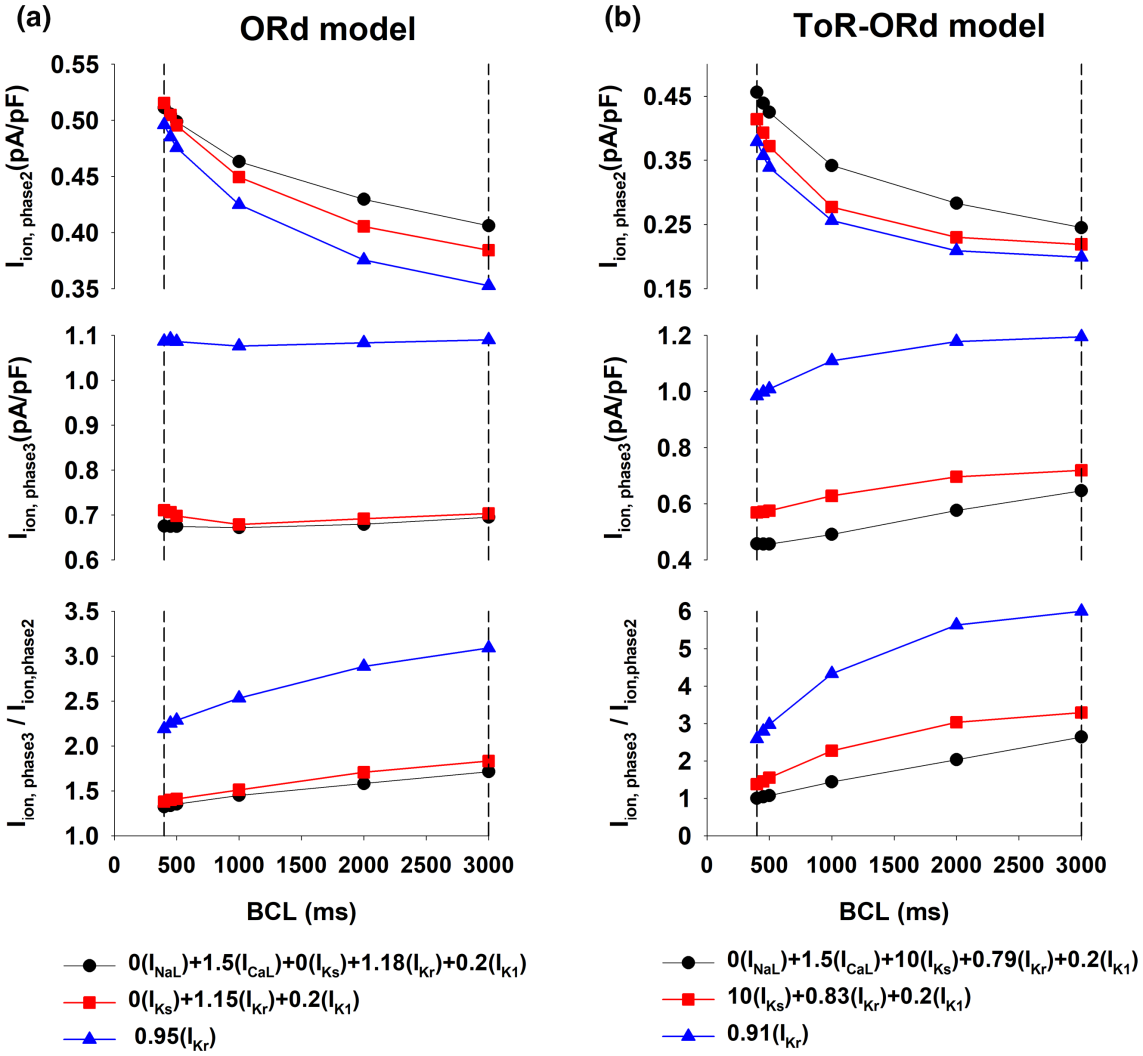
Average I_ion_ during phase 2 (top) and phase 3 (middle) of the action potential as well as their ratio I_ion,phase3_/I_ion,phase2_ (bottom), for different BCLs, using the ORd model (a) and the ToR‐ORd model (b). See text for detailed description.

Figure 4 shows a summary of average I_ion_ during phase 2 and phase 3 as well as their ratio, for different BCLs, for the ORd model (Figure 4a) and the ToR‐ORd model (Figure 4b). APDs for all interventions are the same only with BCL = 3000 ms; for shorter BCLs, APDs varied because different interventions have a different rate dependence (Figure 1). For all BCLs, interventions with a strong positive rate dependence (black circles and red squares) exhibit a larger average I_ion_ during phase 2 (top panel), a smaller average I_ion_ during phase 3 (middle panel), and a smaller I_ion,phase3_/I_ion,phase2_ ratio (bottom panel) than for the intervention with reverse rate dependence (blue triangles up). Figure 4 also shows that a faster stimulation rate (i.e., a shorter BCL) results in a robust increase of average I_ion_ during phase 2, with a moderate (in the ToR‐ORd model) or marginal decrease (in the ORd model) of average I_ion_ during phase 3. Consequently, most of the shortening of the action potential with rate in both models occurs as a result of a shortening of phase 2, with phase 3 remaining relatively constant or slightly increased.

**FIGURE 5:**
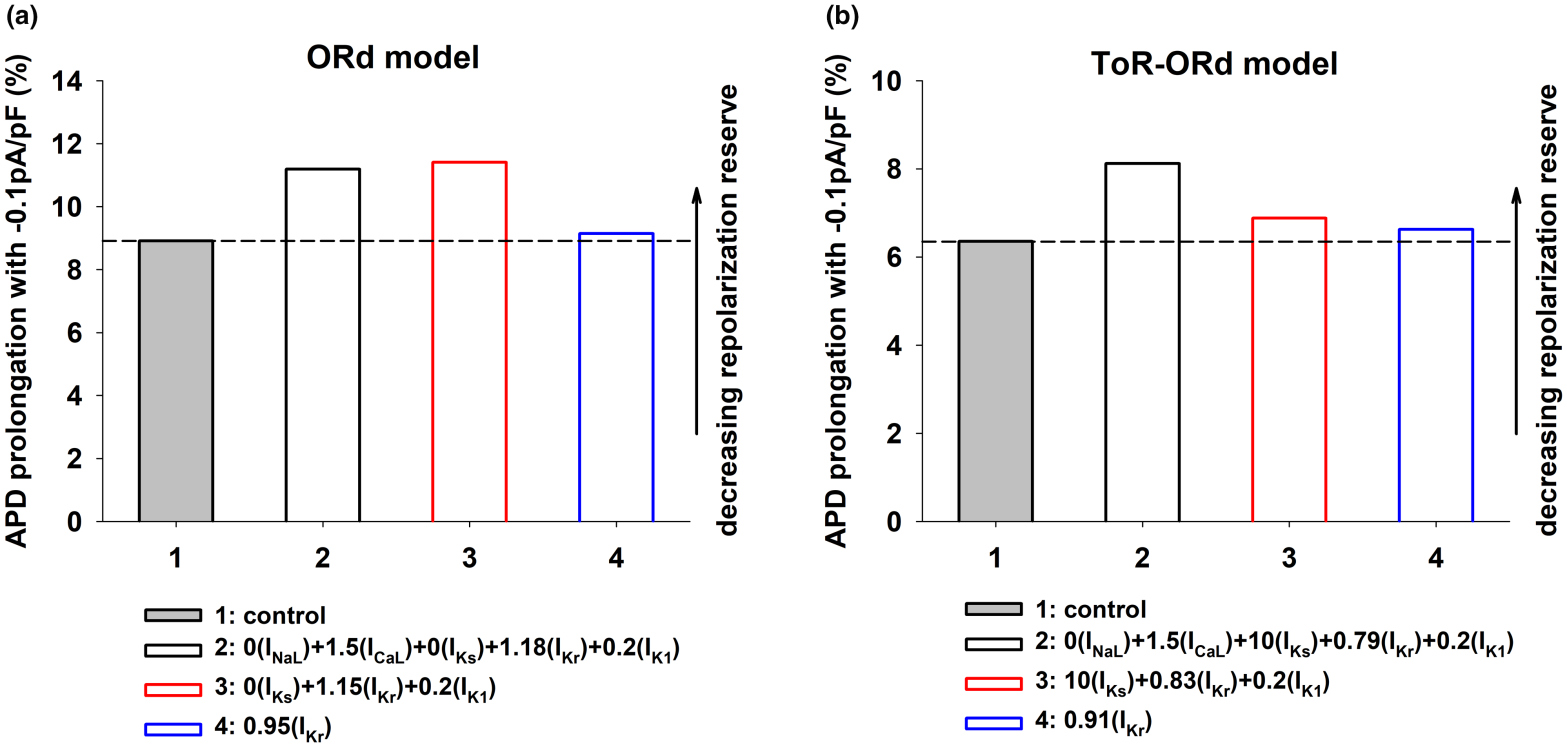
Percentage prolongation of APD when a constant depolarizing current of −0.1pA/pF is applied during the action potential when BCL = 3000 ms for the control action potential (gray bar, dashed horizontal line) and for the three interventions in Figures 2, 3, 4. Panel a: Interventions that result in a positive rate dependence (bars #2 and #3) and a reverse rate dependence (bar#4) using the ORd model. Panel b: Interventions that result in positive rate dependence (bars #2 and #3) and reverse rate dependence (bar #4) using the ToR‐ORd model. For interventions with positive rate dependence (bar #2 and bar #3 in Panels a and b), APD prolongation is above the dashed line indicating that the repolarization reserve is decreased with respect to control. See text for detailed description.

Interventions that prolong the action potential with a positive rate dependence result in a smaller I_ion,phase3_/I_ion,phase2_ ratio. Average I_ion_ over a time interval is the average slope of action potential repolarization during that interval (Cabo, 2022); therefore, a smaller I_ion,phase3_/I_ion,phase2_ ratio indicates a triangulation of the action potential shape (Figures 2 and 3). Action potential triangulation may result in the development of triggered arrhythmias (Hondeghem et al., 2001; Kannankeril et al., 2010; Shah & Hondeghem, 2005). To compare the pro‐arrhythmic risk of the interventions in Figures 1, 2, 3, 4 we estimated their repolarization reserve with BCL = 3000 ms (Figure 5). The figure shows that interventions that result in a positive rate dependence (Figure 5a,b, bars #2 and #3) have a decreased repolarization reserve with respect to control (Figure 5a,b, bar #1). For the ORd model, there is no difference in the repolarization reserve for the intervention that modulates both depolarizing and repolarizing currents (Figure 5a, bar #2) and the intervention that modulates only repolarizing currents (Figure 5a, bar #3). In contrast, for the ToR‐ORd model, the intervention with a stronger positive rate dependent response (Figure 5b, bar #2) has a smaller repolarization reserve that the intervention with a smaller positive rate dependent response (Figure 5b, bar #3).

### 3.3 Relative importance of ion currents to achieve a positive rate‐dependent APD prolongation

**FIGURE 6:**
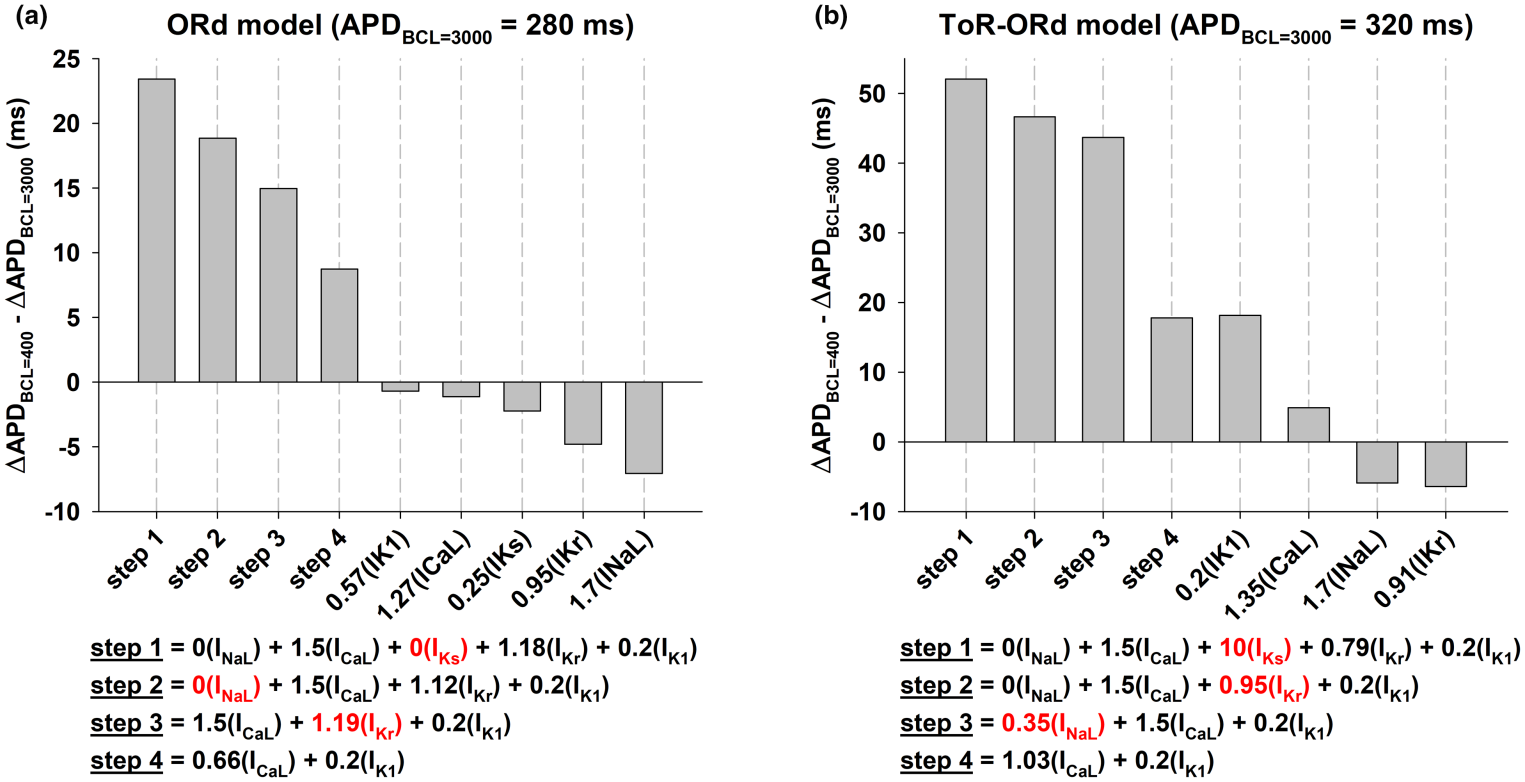
Panel a: Relative importance of each ion current to obtain a positive rate‐dependent APD prolongation to 280 ms, when BCL = 3000 ms, using the ORd model. The figure shows the four steps in the backward feature elimination procedure starting with the intervention that modulates both depolarizing and repolarizing ion currents (0I_NaL_ + 1.5I_CaL_ + 0I_Ks_ + 1.18I_Kr_ + 0.2I_K1_) in Figure 1a (black circles). The interventions that maximize the positive rate‐dependent prolongation to 280 ms after each step (steps 1–4) are shown below the graph. For each step, the ion current that contributes less to positive rate dependence is shown in red and it is eliminated in the next step. The *y*‐axis shows the difference between APD prolongation (with respect to control) when BCL = 400 ms and APD prolongation when BCL = 3000 ms: a positive value indicates positive rate dependence. The figure also shows that the modulation of each channel individually does not result in a positive rate‐dependent APD prolongation to 280 ms in the ORd model. Panel b: Relative importance of each ion current to a positive rate‐dependent APD prolongation to 320 ms, when BCL = 3000 ms, using the ToR‐ORd model. The figure shows the four steps in the backward feature elimination procedure starting with the intervention that modulates both depolarizing and repolarizing ion currents (0I_NaL_ + 1.5I_CaL_ + 10I_Ks_ + 0.79I_Kr_ + 0.2I_K1_) in Figure 1b (black circles). The format is the same as in panel a. The figure also shows that specific modulation of I_CaL_ or I_K1_ results in positive rate dependence in the ToR‐ORd model. See text for detailed description.

The previous results show that the combined modulation of depolarizing currents (I_NaL_ and I_CaL_) and repolarizing currents (I_Ks_, I_Kr_, and I_K1_) results in a robust positive rate dependence (Figure 1a,b, black circles). We used the backward feature elimination procedure to investigate the relative importance of each current to achieve a positive rate‐dependent APD prolongation using the ORd model (Figure 6a) and the ToR‐ORd model (Figure 6b).

Figure 6a shows the four steps in the backward feature elimination procedure for the ORd model when maximizing the positive rate‐dependent response of APD prolongation to 280 ms, with BCL = 3000 ms (Figure 1a, black circles). Step 1 shows the positive rate‐dependent response (=23 ms; estimated as ΔAPD_BCL = 400ms_–ΔAPD_BCL = 3000ms_) when all I_NaL_, I_CaL_, I_Ks_, I_Kr_, and I_K1_ are modulated. We then estimated the positive rate‐dependent response for the five different current combinations that result from eliminating each ion current (see Section 2 above). The ion current combination that results in the largest decrease in the positive rate‐dependent response (from 23 to 12 ms) is [I_NaL_, I_CaL_, I_Ks_ and I_Kr_], indicating that I_K1_ is the current contributing the most to a positive rate‐dependent response in step 1. The current combination that results in the smallest decrease in the positive rate‐dependent response (from 23 to 19 ms) is [I_NaL_, I_CaL_, I_Kr_ and I_K1_], indicating that I_Ks_ is the current contributing the least to a positive rate‐dependent response in step 1. Therefore, I_Ks_ is eliminated as the elimination procedure progresses from steps 1 to 2, and its maximum conductance is set to the control value. Note that elimination of I_Ks_ results in a 17% decrease (from 23 to 19 ms) in the positive rate‐dependent response, indicating that, even though I_Ks_ is the current that contributes less to a positive rate‐dependent response in step 1, its contribution is still important. Using the same procedure, I_NaL_ and I_Kr_ are eliminated in steps 3 and 4 respectively, with the consequent decrease in positive rate dependence. Since all interventions in steps 1 to 4 result in the same APD prolongation to 280 ms, the progressive elimination of currents from step to step leads to an adjustment of the maximum conductances of the remaining currents. For example, the elimination of I_Kr_ is step 3 (i.e., setting its maximum conductance to the control value) implies a reduction of the maximum conductance of I_Kr_ from 1.19× to 1× the control value in step 2, which, if not compensated, would prolong APD. That prolongation is compensated by a reduction of the maximum conductance of I_CaL_ from 1.5× to 0.66× the control value, which shortens APD, to maintain APD = 280 ms. The backward feature elimination procedure identifies I_CaL_ and I_K1_ as the ion currents that are more important for positive rate dependence. Figure 6a also shows that selective modulation of specific ion currents does not result in a positive rate‐dependent APD prolongation in the ORd model. However, selective modulation of I_K1_ results in a smaller reverse rate‐dependent APD prolongation than selective modulation of other ion currents.

Figure 6b shows the four steps in the backward feature elimination procedure for the ToR‐ORd model when maximizing the positive rate‐dependent response of APD prolongation to 320 ms, with BCL = 3000 ms (Figure 1b, black circles). The combination that results in the largest decrease in the positive rate dependent response (from 52 to 21 ms) is [I_NaL_, I_CaL_, I_Ks_ and I_Kr_], indicating that I_K1_ is the current contributing the most to a positive rate‐dependent response in step 1. The combination that results in the smallest decrease in the positive rate‐dependent response (from 52 to 47 ms) is [I_NaL_, I_CaL_, I_Kr_ and I_K1_], indicating that I_Ks_ is the current contributing the least to a positive rate‐dependent response in step 1. Therefore, as it happened with the ORd model, I_Ks_ is eliminated as the elimination procedure progresses from step 1 to step 2, and its maximum conductance is set to the control value. Using the same procedure, I_Kr_ and I_NaL_ are eliminated in steps 2 and 3 respectively, with the consequent decrease in positive rate dependence. Note that there is a 59% (from 44 to 18 ms) decrease in the positive rate‐dependent response with the elimination of I_NaL_ from steps 3 to 4. That large decrease indicates that I_NaL_ is an important contributor to a positive rate‐dependent response; but the decrease would be larger if either I_CaL_ or I_K1_ were eliminated in step 3. As it happened with the ORd model, the two most important currents for a positive rate‐dependent response, when the backward feature elimination procedure reaches step 4, are I_CaL_ and I_K1_. As we mentioned earlier, the ToR‐ORd model shows a more robust positive rate dependence than the ORd model. In contrast to the ORd model, selective modulation of I_K1_ and I_CaL_ result in a positive rate dependence response, with I_K1_ showing a more robust positive rate‐dependent response than I_CaL_. Figure 6b does not show the effect of I_Ks_ block on positive rate dependence because, given the small contribution of I_Ks_ to action potential repolarization in the ToR‐ORd model, complete block of I_Ks_ was not enough to prolong APD to 320 ms.

### 3.4 APD prolongation of different magnitudes with a positive rate dependence

**FIGURE 7:**
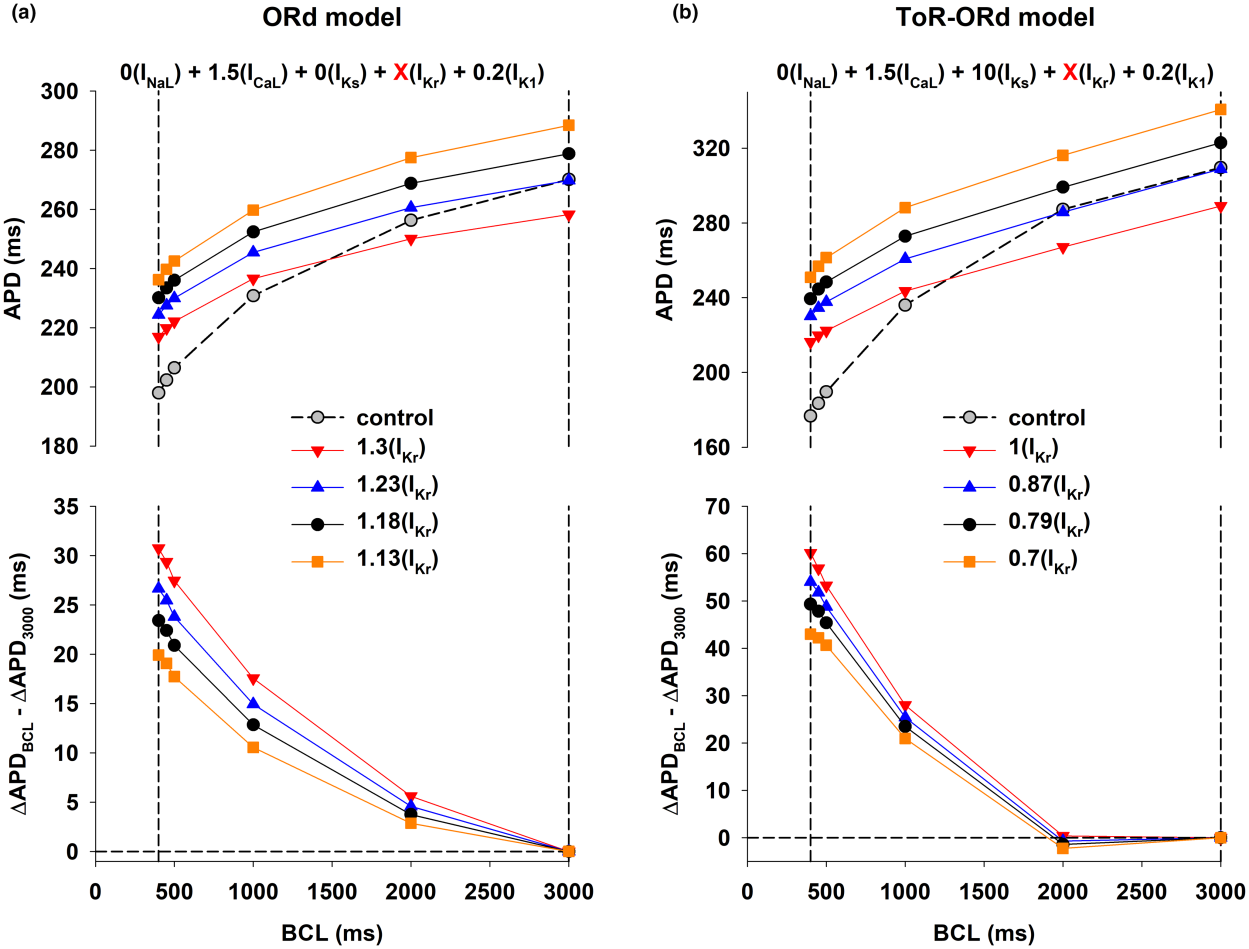
Interventions causing different amounts of APD prolongation with a positive rate dependence. Panel a (top): APD rate dependence with the ORd model for control (dashed black line) and four interventions with fixed modulation of I_NaL_, I_CaL_, I_Ks_ and I_K1_, and varying degrees of I_Kr_ modulation (0I_NaL_ + 1.5I_CaL_ + 0I_Ks_ + *X*I_Kr_ + 0.2I_K1_), with *X* = 1.13 (ochre squares), 1.18 (black circles), 1.23 (blue triangles up) and 1.3 (red triangles down). Panel a (bottom): Difference between APD prolongation (with respect to control) for each BCL and APD prolongation with BCL = 3000 ms for the four interventions in Panel a (top). A positive value indicates positive rate dependence. The vertical dashed lines indicate BCL = 400 ms and 3000 ms. Panel b shows equivalent results with the ToR‐ORd model using the same format. See text for detailed description.

The modulation of depolarizing and repolarizing ion currents in Figures 1, 2, 3, 4, 5, 6 produced an APD prolongation of about 10 ms with respect to control at a BCL = 3000 ms. Varying the modulation of I_Kr_ while keeping modulation of the other ion currents at the optimal values that resulted in positive rate‐dependent response makes it possible to produce different amounts of APD prolongation (Figure 7). Figure 7a (top) shows the rate‐dependent responses of control (dashed black line) and 4 different modulations (activations) of I_Kr_ resulting in different positive rate‐dependent APD prolongations in the ORd model. Indeed, it is possible to prolong APD at fast stimulation rates (BCL = 400 ms) with no APD prolongation (*X* = 1.23I_Kr_, blue triangles up in Figure 7a, top) or with APD shortening (*X* = 1.3I_Kr_, red triangles down in Figure 7a, top) at slow stimulation rates (BCL = 3000 ms). Figure 7a (bottom) shows that larger APD prolongations results in a decrease in the positive rate‐dependent response. For example, the larger APD prolongation (*X* = 1.13I_Kr_, ochre squares in Figure 7a, top) leads to the smaller rate‐dependent response (*X* = 1.13I_Kr_, ochre squares in Figure 7a, bottom). Figure 7b shows similar results for the ToR‐ORd model.

**FIGURE 8:**
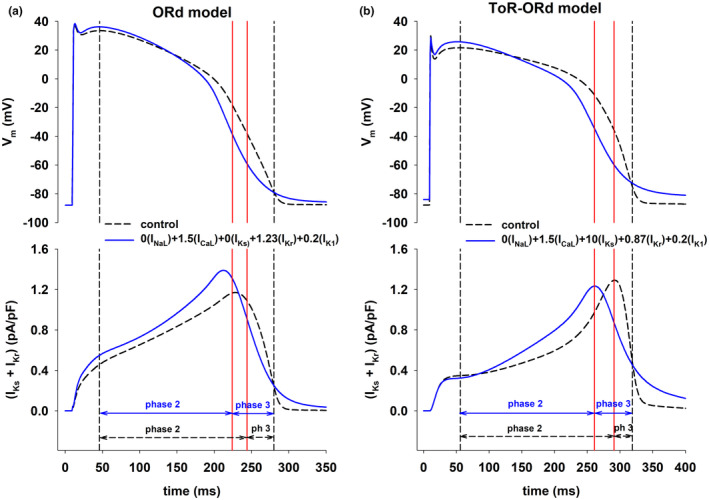
Dynamics of the total delayed rectifier potassium currents (= I_Ks_ + I_Kr_) for control action potentials and action potentials with positive rate dependence. Panel a (top): Control action potential (black dashed line) and action potential with positive rate dependence (solid blue line) with the same APD during stimulation with BCL = 3000 ms, with the ORd model. Panel a (bottom): I_Ks_ + I_Kr_ during the action potentials in the top of panel a. The leftmost vertical dashed line indicates the beginning of phase 2 repolarization; the rightmost vertical dashed line indicates the time of 90% repolarization. The vertical red solid lines indicate the end of phase 2 (and beginning of phase 3) for both action potentials. The duration of phase 2 and 3 are indicated by the arrows. Panel b shows equivalent results for the ToR‐ORd model, and it has the same format as panel a. See text for detailed description.

The optimal combination of ion current modulation to achieve a positive rate‐dependent response requires the use I_Ks_ blockers and I_Kr_ activators in the ORd model (Figure 7a), but the use of I_Ks_ activators and I_Kr_ blockers in the ToR‐ORd model (Figure 7b); those differences are a consequence of the different formulations of I_Ks_ and I_Kr_ in the models. Figure 8 shows a control action potential (Figure 8, top, dashed black line) and an action potential with a positive rate‐dependent response (Figure 8, top, solid blue line) having the same APD with BCL = 3000 ms (blue triangles up and gray circles in Figure 7). The figure shows that the dynamics of the combined effect of I_Ks_ + I_Kr_ during repolarization are similar for both models (Figure 8a,b, bottom): an increase of delayed rectifier currents (i.e., I_Ks_ + I_Kr_) during phase 2 of the action potential and a decrease of delayed rectifier currents during phase 3 are associated with a positive rate dependent response.

**FIGURE 9:**
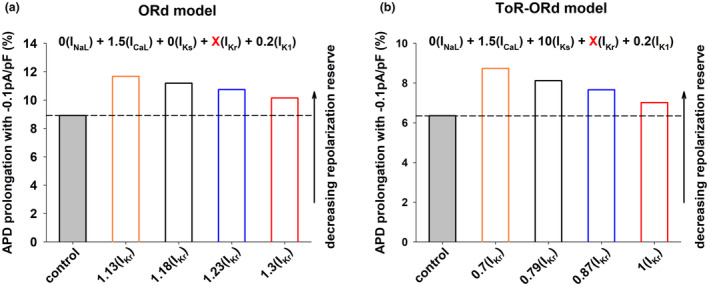
Percentage prolongation of APD when a constant depolarizing current of −0.1pA/pF is applied during the action potential when BCL = 3000 ms for the control action potential (gray bar, dashed line) and for the interventions in Figure 7. Panel a: Interventions that result in a positive rate dependence using the ORd model (0I_NaL_ + 1.5I_CaL_ + 0I_Ks_ + *X*I_Kr_ + 0.2I_K1_, where *X* is the value in the *x*‐axis). Panel b: Interventions that result in a positive rate dependence using the ToR‐ORd model (0I_NaL_ + 1.5I_CaL_ + 10I_Ks_ + *X*I_Kr_ + 0.2I_K1_, where *X* is the value in the *x*‐axis). For all interventions, APD prolongation is above the dashed line indicating that the repolarization reserve is decreased with respect to control. See text for detailed description.

Figure 9 shows the repolarization reserve with BCL = 3000 ms for the interventions in Figure 7 with respect to the control action potential (gray bar, dashed horizontal line). For both models, the larger the APD prolongation (ochre squares in Figure 7a,b), the smaller the repolarization reserve (*X* = 1.13I_Kr_, ochre bars in Figure 9a,b). Note that for interventions that prolong APD with BCL = 400 ms but shorten it with BCL = 3000 ms (red triangles down in Figure 7a,b), the repolarization reserve is just slightly decreased over that of control (*X* = 1.3I_Kr_ in Figure 9a and *X* = 1I_Kr_ in Figure 9b). The results in Figure 9 suggest that multichannel modulation of depolarizing and repolarizing currents that prolong APD at fast heart rates (close to those occurring during ventricular tachycardia) and shorten APD at slow heart rates should minimize the risk of triggered arrythmias.

### 3.5 Relationship between total ion current and APD during repolarization

**FIGURE 10:**
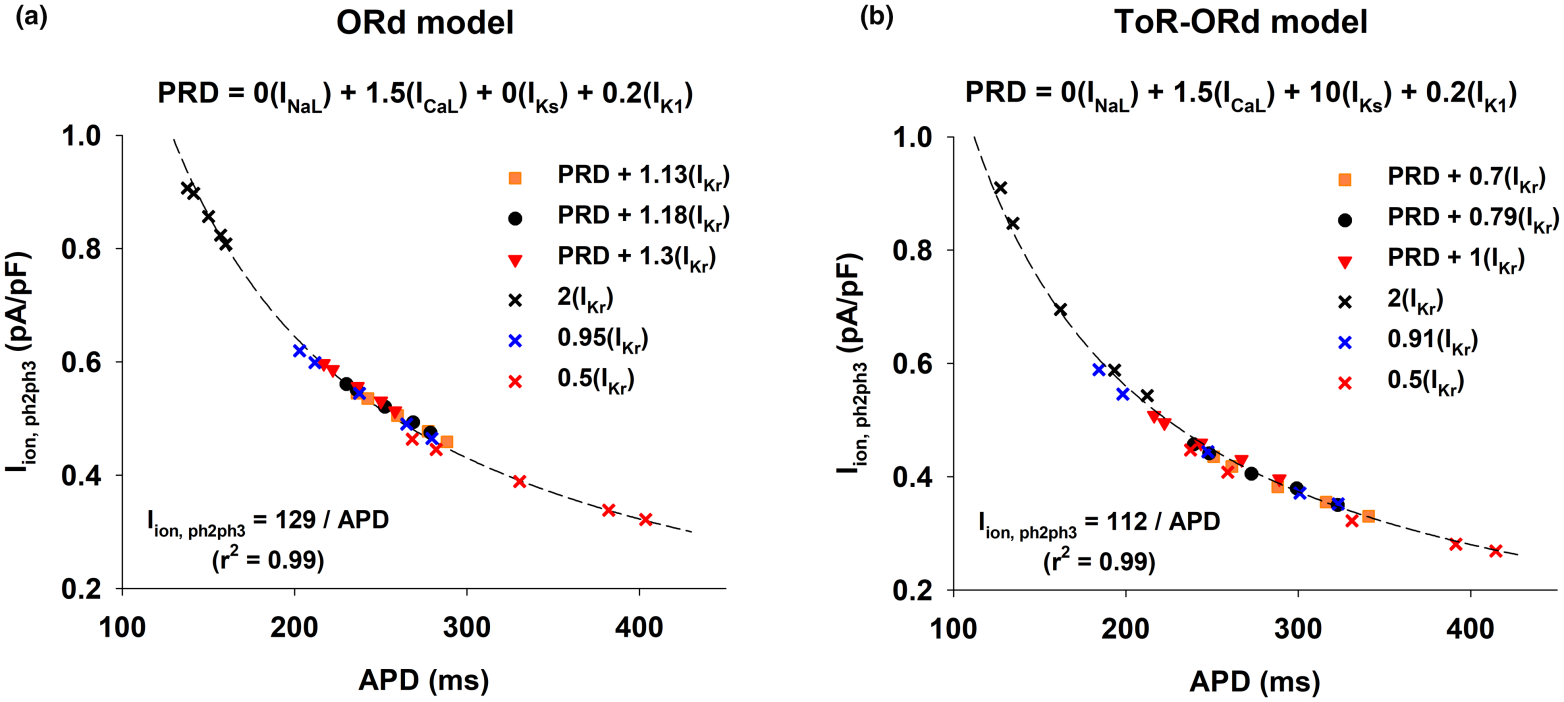
Relationship between average total I_ion_ during phase 2 and phase 3 (I_ion,ph2ph3_) and APD for different interventions that prolong or shorten (black Xs) the control APD, using the ORd model (a) and the ToR‐ORd model (b). In both models, the relationship is non‐linear, and it can be modeled by a hyperbola, I_ion,ph2ph3_ = K/APD, where K is the approximate transmembrane potential change during repolarization, with *r* ^2^ = 0.99. In two of the interventions (blue and red Xs) block of I_Kr_ leads to a reverse rate‐dependent APD prolongation. In three of the interventions (ochre squares, black circles and red triangles down, which correspond to the same symbols/colors in Figure 7) multichannel modulation of depolarizing and repolarizing currents leads to a positive rate‐dependent APD prolongation. Interventions labeled PRD + *X*(I_Kr_) represent interventions with fixed modulation of I_NaL_, I_CaL_, I_Ks_ and I_K1_, and varying degrees of I_Kr_ modulation: (0I_NaL_ + 1.5I_CaL_ + 0I_Ks_ + XI_Kr_ + 0.2I_K1_) in (a), and (0I_NaL_ + 1.5I_CaL_ + 10I_Ks_ + XI_Kr_ + 0.2I_K1_) in (b). See text for detailed description.

Figure 10 shows the relationship between average total I_ion_ during phase 2 and phase 3 (I_ion,ph2ph3_) and APD for different interventions that prolong or shorten the control APD, using the ORd model (Figure 10a) and the ToR‐ORd model (Figure 10b). In both models, for all interventions, the relationship is non‐linear, and it can be modeled by a hyperbola (I_ion,ph2ph3_ = K/APD; where K is the approximate transmembrane potential change during repolarization; dashed line in Figure 10), in agreement with Banyasz et al. (2009). In two of the interventions in Figure 10a,b (blue and red Xs) block of I_Kr_ leads to a reverse rate‐dependent APD prolongation. In three of the interventions multichannel modulation of depolarizing and repolarizing currents leads to a positive rate‐dependent APD prolongation (squares, circles and triangles down in Figure 10a,b; the positive rate‐dependence for those interventions is shown in Figure 7). For both, reverse and positive rate‐dependent interventions, the relationship between I_ion,ph2ph3_ and APD is non‐linear (Figure 10). Banyasz et al. (2009) proposed that reverse rate‐dependence could be a consequence of the non‐linear relationship between I_ion_ and APD during repolarization. Our results show that interventions resulting in a positive rate‐dependent ADP prolongation also exhibit a non‐linear relationship between I_ion_ and APD, suggesting that such a non‐linear relationship may not be limited to interventions with reverse rate‐dependent APD prolongation (Figure 10).

## 4 DISCUSSION

We have shown that, in computer models of the human ventricular action potential, the combined modulation of both depolarizing and repolarizing ion currents (I_NaL_, I_CaL_, I_Ks_, I_Kr_ and I_K1_) results in a stronger positive rate dependent APD prolongation than modulation of just repolarizing potassium currents (I_Ks_, I_Kr_ and I_K1_); selective modulation of a specific repolarizing current (I_Kr_) results in reverse rate dependence (Figures 1, 2, 3). A strong positive rate‐dependent APD prolongation correlates with an acceleration of phase 2 repolarization and a deceleration of phase 3 repolarization, which leads to a triangulation of the action potential shape (Figure 4). A positive rate dependent APD prolongation decreases the repolarization reserve at slow excitation rates (Figure 5), which can be managed by interventions that prolong APD at fast excitation rates and shorten APD at slow excitation rates (Figures 7 and 9). Feature importance analysis shows that, in both the ORd and ToR‐ORd models, I_CaL_ and I_K1_ are the most important ion currents to achieve a positive rate‐dependent APD prolongation (Figure 6).

There is experimental and clinical evidence showing that selective block of a specific potassium current results in reverse rate‐dependent APD prolongation (Banyasz et al., 2009; Barandi et al., 2010; Dorian & Newman, 2000; Virag et al., 2009). Moreover, Banyasz et al. (2009) and Barandi et al. (2010) demonstrated reverse rate‐dependence with a variety of pharmacological agents aimed at specific ion channels that either prolonged or shortened APD, suggesting that reverse rate‐dependence may be an intrinsic property of canine myocardium, and that a positive rate‐dependent APD prolongation may be unattainable. The selective modulation of specific ion channels also results in a reverse rate‐dependent APD prolongation in the ORd model (Figure 6a) or in a small positive rate‐dependent response in the ToR‐ORd model (Figure 6b, specific modulation of I_K1_ or I_CaL_). Moreover, our results show that optimal modulation of several depolarizing and repolarizing ion currents results in a robust positive rate‐dependent APD prolongation (Figures 1, 2, 3, 4, 5, 6). It is possible that the pharmacological agents tested by Banyasz et al. (2009) do not provide the optimal multichannel modulation (acceleration of phase 2 repolarization and deceleration of phase 3 repolarization, Figures 2, 3, 4) needed for a positive rate‐dependent APD prolongation. Alternatively, it is possible that the computer models of the action potential of single human ventricular epicardial cells used in this report might not adequately reproduce all rate‐dependent effects of the multicellular canine right ventricular preparations used in Banyasz et al. (2009).

There is also abundant clinical and experimental evidence suggesting that the combined modulation of several ion currents may result in neutral (neither positive nor reverse) rate‐dependent APD prolongation (Bril et al., 1995, 1998; Dorian & Newman, 2000; Faivre et al., 1999; Hondeghem & Snyders, 1990; Kassotis et al., 2003; Lathrop & Varró, 1989, 1990; Mátyus et al., 2004; Nadler et al., 1998; Qi et al., 1999; Shibata et al., 2012; Varga et al., 2021; VerNooy & Mangrum, 2005). The exact mechanism underlying the attenuation of reverse rate dependence by the combined modulation of several ion currents is unknown. In that context, our results suggest a strategy on how multichannel pharmacology can produce a robust positive rate‐dependent APD prolongation by optimal modulation of both depolarizing and repolarizing ion currents, using ion channel activators and blockers, which should result in an antiarrhythmic effect.

We have shown earlier (Cabo, 2022) that the combination of potassium channel activators and blockers can produce a positive rate dependent APD prolongation in two different models of the human ventricular action potential: the TNNP model (ten Tusscher et al., 2004) and the ToR‐ORd model (Tomek et al., 2019). Despite differences in the formulation of major repolarizing currents in the models (for example, the TNNP model has an unphysiologically large I_Ks_) both models predict a positive rate‐dependent APD prolongation by enhancing I_Ks_ and blocking I_Kr_ and I_K1_. In this report, we show that optimal modulation of those same three potassium currents also results in positive rate‐dependent APD prolongation in the ORd model (O'Hara et al., 2011). However, in contrast to the other two models, positive rate dependence in the ORd model requires complete block of I_Ks_ (instead of I_Ks_ enhancement), enhancement of I_Kr_ (instead of I_Kr_ inhibition) and I_K1_ block. Despite differences in the formulation of the ion currents, the dynamics of the total delayed rectifier current (i.e., I_Ks_ + I_Kr_) are similar in both models and further support the idea that an acceleration of phase 2 and deceleration of phase 3 repolarization of the action potential is associated with a positive rate‐dependent response (solid blue line in Figure 9).

Experimental evidence on whether I_K1_ inhibition results in positive or reverse rate dependence is mixed. Positive rate dependence has been demonstrated in guinea‐pig myocytes treated with I_K1_ blocker terikalant (Williams et al., 1999), while inhibition of I_K1_ with either terikalant (Biliczki et al., 2005) or BaCl_2_ (Banyasz et al., 2009; Virag et al., 2009) in canine myocytes results in reverse rate dependence. The results are also mixed in computer models: specific I_K1_ block does not result in a positive rate‐dependent response in the ORd model (I_K1_ in Figure 6a), but it results in a positive rate‐dependent response in the ToR‐ORd model (I_K1_ in Figure 6b). Earlier numerical studies also suggested the importance of I_K1_ for a positive rate‐dependent response (Cummins et al., 2014). Several findings in this report, which are consistent in both models, point to the importance of I_K1_ to achieve a positive rate‐dependent APD prolongation: (1) Elimination of I_K1_ in step 1 of the backward feature elimination procedure results in the largest decrease of the positive rate‐dependent response (estimated as ΔAPD_BCL = 400_‐ΔAPD_BCL = 3000_ in Figure 6) when compared to the elimination of other ion currents; (2) Step 4 of the backward feature elimination procedure suggests that I_K1_ is the most important repolarizing current to obtain a positive rate dependent response (step 4 in Figure 6a,b); (3) When comparing the effect of modulation of individual currents on positive rate dependence (Figure 6), I_K1_ block results in the largest positive rate‐dependent response in the ToR‐ORd model or in the smallest reverse rate‐dependent response in the ORd model. All in all, based on the available experimental and numerical simulation evidence, it is unclear that specific block of I_K1_ would result in a positive rate‐dependent APD prolongation; but our results suggest that including block of I_K1_ as part of a multichannel modulation intervention would result in a more robust positive rate‐dependent APD prolongation.

Chronic amiodarone and ranolazine (Belardinelli et al., 2006), which block I_NaL_, I_Ca_, I_Ks_ and I_Kr_, exhibit a neutral (neither positive nor reverse) rate dependent APD prolongation, in contrast with the reverse rate‐dependent APD prolongation of Class III agents that block a specific ion channel type like I_Kr_. This multichannel inhibition profile has been linked to their anti‐arrhythmic efficacy (Antzelevitch et al., 2004). The neutral rate‐dependent APD prolongation of amiodarone and ranolazine is associated with block of depolarizing and repolarizing ion currents, which has two opposing effects on APD: block of I_NaL_ and I_Ca_ tend to shorten APD; block of I_Ks_ and I_Kr_ tend to lengthen APD. In our simulations, a robust positive rate dependence response also requires modulation of depolarizing and repolarizing ion currents. The strong positive rate‐dependent APD prolongation shown in Figure 1 (black circles) is also associated with two opposing effects of depolarizing and repolarizing currents on APD, but, in contrast to amiodarone and ranolazine, those effects are achieved by the use of ion channel activators instead of ion channel blockers: enhancement of I_CaL_ tends to lengthen APD; enhancement of the total delayed rectifier current tends to shorten APD. The results in Figure 1 further suggest that modulation of multiple ion channels with activators and blockers (Figure 1, black circles) can produce a more robust positive rate dependence than modulation of those same ion channels with just ion channel blockers (Figure 1, green triangles down) at fast excitation rates (BCLs <500 ms). I_Ks_ activators (Tamargo et al., 2004; Xu et al., 2002, 2015) and I_Kr_ activators (Shi et al., 2020) have been developed to prevent excessive APD prolongation that may occur in patients suffering LQT syndrome, cardiac hypertrophy or cardiac failure. I_CaL_ activators have been shown to prevent reentrant arrhythmias in experimental models of myocardial infarction (Cabo et al., 2000).

The multichannel inhibition profile of amiodarone and ranolazine has also been linked to their lack of proarrhythmic effects (Antzelevitch et al., 2004). Block of depolarizing currents like I_NaL_ and I_CaL_ result in acceleration of phase 2 repolarization, preventing the initiation of triggered arrhythmias that may result in Torsade de Pointes. In our simulations, a positive rate‐dependent APD prolongation is also associated with an acceleration of phase 2 repolarization (Figure 4). However, the mechanism of that acceleration is different: the inhibition of I_NaL_ and the enhancement of the delayed rectifier currents (I_Ks_ + I_Kr_) compensate the enhancement of I_CaL_. While acceleration of phase 2 repolarization can reduce the likelihood of triggered arrhythmias, a strong positive rate‐dependent APD prolongation in our simulations is associated with a decrease in the repolarization reserve with respect to control action potentials (Figures 5 and 9), which could indicate a pro‐arrhythmic risk. The decrease in the repolarization reserve can be managed by limiting the amount of I_K1_ inhibition. Interventions with moderate I_K1_ block (≤50%) still result in a robust positive rate response with moderate decrease in the repolarization reserve with respect to control (Cabo, 2022). Additionally, interventions that prolong APD at fast excitation rates, but shorten APD at slow excitation rates further moderate the decrease in the repolarization reserve and the risk of initiation of triggered arrhythmias (Figure 7, red triangles down; Figure 9, red bar).

In conclusion, multichannel modulation of depolarizing (I_NaL_ and I_CaL_) and repolarizing currents (I_Ks_, I_Kr_ and I_K1_), with ion channel activators and blockers, results in a robust APD prolongation at fast excitation rates, which should be anti‐arrhythmic, while minimizing APD prolongation at slow heart rates, which should reduce potential pro‐arrhythmic risks.

### 4.1 Limitations

Computer models of the action potential integrate, often conflicting, experimental data obtained under different conditions from different preparations. As a consequence, conclusions derived from numerical simulations of the cardiac action potential should be interpreted with caution as predictions that need to be tested experimentally. In this report we simulate the effect of anti‐arrhythmic agents on ion channels by modulating the maximum channel conductance (Section 2). However, pharmacological agents have binding and unbinding kinetics which may change at different stimulation rates, adding another layer of complexity to their modulation of the channel which was not considered in this report.

The ORd and ToR‐ORd models used in this report simulate healthy ventricular action potentials. But the density and kinetics of ion channels of myocytes is remodeled by disease (Baba et al., 2005), and the effect of drug agents on remodeled myocytes may be different from their effect in healthy myocytes (Cabo & Boyden, 2003). Therefore, the conclusions of our simulations may not apply to diseased myocardial cells. Moreover, during the propagation of the action potential in myocardial tissue, electrotonic interaction between neighboring cells can modulate the effects of enhancing or inhibiting ion currents observed in single cells (Decker et al., 2009). Therefore, to elucidate how positive rate dependent APD prolongation affects the dynamics of propagation of premature impulses that initiate and sustain reentrant arrhythmias in heterogeneous and possibly remodeled myocardial tissue requires additional computational and experimental studies.

## FUNDING INFORMATION

This work was supported in part by PSC‐CUNY Award # 65033‐0053.

## CONFLICT OF INTEREST STATEMENT

The author declares that there is no conflict of interest.

## ETHICAL STATEMENT

This study does not require ethical approval.
